# Outage Analysis of Parasitic Ambient Backscatter Communication in Decode-and-Forward Relay Networks with SWIPT

**DOI:** 10.3390/s20051273

**Published:** 2020-02-26

**Authors:** Yanhong Tuo, Chao Zhang

**Affiliations:** School of Information and Communications Engineering, Xi’an Jiaotong University, Xi’an, Shaanxi 710049, China; tuoyanhong@stu.xjtu.edu.cn

**Keywords:** simultaneous wireless information and power transfer, ambient backscatter communication, decode-and-forward, successive interference cancellation, outage probability

## Abstract

In this paper, we investigate the outage performance of simultaneous wireless information and power transfer (SWIPT) based Decode-and-Forward (DF) relay networks, where the relay needs to simultaneously forward information for two relaying links, primary relaying link and parasitic relaying link. The primary relaying link is the traditional source-relay-destination relay system. While in the parasitic relaying link, the parasitic source, i.e., Internet-of-Things (IoT) tag, is not connected to the stable power source and thus has to backscatter the signals from the primary source to convey its information. The relay not only harvests energy from Radio Frequency (RF) signals from both sources but also forwards messages to their corresponding destinations. The primary source and destination are unaware of the parasitic backscatter transmission, but the relay and parasitic destination can employ successive interference cancellation (SIC) detector to eliminate the interference from the primary link and detect the message from the parasitic source. In order to investigate the interplay between the primary and parasitic relaying links, the outage probabilities of both relaying links are derived. Besides, the effects of system parameters, i.e., power splitting coefficient, forwarding power allocation coefficient and backscatter reflection coefficient, on the system performance are discussed. Simulation results verify our theoretical analysis. In the meanwhile, it is revealed that the advised relaying system has far larger sum throughput than the one with only primary relaying link and the parasitic relaying link can gain considerable throughput at the cost of negligible degradation of primary throughput.

## 1. Introduction

Backscatter communication enables information transmission by reflecting incident radio frequency (RF) signal and as such enjoys trivial power consumption and low implementation cost. It has been recognized as a promising solution to the massive communication requirements of internet of things (IoT) [1,2,3,4]. Generally speaking, at present there are three main ways to implement backscatter communications, i.e., monostatic, bistatic, and ambient modes [4,5]. In monostatic and bistatic modes, a dedicated carrier transmitter is required to emit excitation RF signal to the backscatter tag. The difference between both modes is that in the monostatic mode the carrier transmitter and information receiver are equipped at the same node and can share the same antenna, and in the bistatic mode carrier transmitter and receiver belong to separate nodes. On the other hand, in ambient backscatter communication (AmBC) mode, the backscatter tag alternatively employs the ambient RF signal as the excitation signal. As IoT is envisioned to provide pervasive and ubiquitous interconnections among various kinds of entities, one of the most significant features of IoT is heterogeneous and compatible with existing networks [6]. For this reason, AmBC can make full use of a mess of various RF signals from heterogeneous wireless networks and has been widely investigated by the research community. To this end, we also focus on AmBC in this paper.

The backscatter tag modulates information onto the incidence RF signal and reflects it to its receiver/reader. The basic principle of backscatter communication is utilizing impedance mismatch to reflect RF signal. Thus, the characteristics of the incidence RF signal can be modified by the tag to convey its information to be transmitted. For examples, On-Off Keying (OOK) [7], Binary Phase Shift Keying (BPSK) [8], Multi-Phase Shift Keying (MPSK) [9], Differential Phase Shift Keying (DPSK) [10], Quadrature Amplitude Modulation (QAM) [11], Multi-frequency-shift Keying (MFSK) [12,13] etc., have been proposed for backscatter communication. Additionally, the Orthogonal Frequency Division Multiplexing (OFDM) signal is exploited to perform AmBC [14,15]. Various detection methods are addressed in [16,17,18,19]. For all these backscatter schemes, the backscattered signal is excited by the incident RF signal. Therefore, except in FSK backscatter scheme, the backscattered signal and ambient RF signal may interfere with each other in certain instances, for example, the tag is close to the legacy receiver. As the FSK backscatter scheme needs extra frequency resources and may not be suitable for massive IoT networks [3], the ambient RF and backscattered signals have the same frequency in our paper.

Along with the rapid development of backscattering technology, AmBC has been applied widely in many wireless systems and incorporated with other advanced wireless technologies. Multi-antenna backscatter tags were considered in order to gain spatial diversity [18,19,20,21,22,23]. The scenarios of multiple backscatter tags were investigated by [5,24,25,26]. In order to prevent the reflected signal from degrading the transmissions of ambient RF signals, the cognitive backscatter tag was proposed in [27,28,29,30]. To improve the energy efficiency of backscatter communication, full-duplex communication was also introduced into backscatter tags [31,32]. In order to achieve high energy efficiency, multiple backscatter tags performing non-orthogonal multiple access (NOMA) was proposed in [33]. Instinctively, the cooperative relaying communications were also incorporated with AmBC. Since the backscatter tag can transmit its information through reflecting incident ambient RF signal, it also can reflect the ambient RF signal to the legacy receiver to improve the reliability of legacy transmission. Then AmBC based relay systems were proposed by [34,35,36]. In [34,35], the ambient RF signal carried no information and the interference from the ambient RF signal was not considered in the AmBC. While the performance of the legacy receiver was not investigated and the non-coherent receiver was employed in [36]. The optimal resource allocation with instantaneous channel state information was addressed for relay-assisted AmBC system in [37]. Based on the two-hop RF signals, the AmBC between relay and the cognitive tag was proposed in [38]. Although the interference from AmBC tag was considered in the primary destination, the secondary AmBC receiver was not interfered by the primary transmission.

Specifically, AmBC naturally causes interference to ambient RF sources. It is of importance to explore interaction and coexistence issues between AmBC and ambient wireless transmissions [39]. Liang et al. in [17,40,41,42] proposed the concept of symbiotic radio to capture the key feature of AmBC and investigated the overall performance of the symbiotic system which incorporates AmBC and other wireless systems. In [17], three practical symbiotic radio schemes, commensal, parasitic, and competitive schemes, were proposed to represent the symbiotic relationship between AmBC and primary transmission. In [40], joint optimal primary transmit power and the reflection coefficient of AmBC tag was studied in different symbiotic setups. A full-duplex AmBC tag in the parasitic scheme was proposed in [41], where the optimization resource allocation was addressed. Furthermore, the symbiotic system consisting of AmBC and NOMA system was proposed in [42,43]. From a practical viewpoint, it is costly to let the IoT backscatter tag fully cooperate with the ambient RF transmitter due to signaling overhead. Considering the transmission quality of the authorized RF signal, the full competition from massive IoT tags is not allowed. Thus, the parasitic AmBC, which means we could only improve the performance of IoT AmBC transmission under the condition that the performance of primary transmission is guaranteed or the performance degradation generated by AmBC is acceptable.

In this paper, we investigate the symbiotic relaying system of AmBC and DF relay networks using SWIPT. To the best of our knowledge, it is the first work concerning the symbiotic relaying system. In our considered symbiotic radio system, there are two source-destination pairs, primary source-destination pair and parasitic source-destination pair. Both pairs have no direct transmission channel, so the DF relay is designed to help both sources to forward messages for their corresponding destinations. Considering the IoT scenario, we assume the relay is not connected to a stable power supply and has to harvest energy from the ambient RF signal. Thus, the DF relay adopts the SWIPT during the source transmission phase. The power splitting structure is adopted at the relay. Thus, the relay can detect the received information signal while harvesting energy (refer to [44] and references therein). If the harvested energy can cover the circuit dissipation, the relay performs DF relaying scheme with the left harvested energy. Furthermore, the relay needs to forward two different messages to both destinations simultaneously, therefore, the power domain NOMA is employed to broadcast regenerated signals. The parasitic relaying transmission is fully compatible with the traditional DF relay scheme. In order to investigate the interplay between primary and parasitic relaying links, we derive the analytical expressions of outage probabilities and throughput of both relaying links. Besides, the effects of system parameters on the system performance are discussed via our theoretical results. Finally, the simulation results verify our theoretical results. We also show that the advised relaying system has far larger sum throughput than the one with only primary relaying link and the parasitic relaying link can gain considerable throughput at the cost of negligible degradation of primary throughput. The main contributions of this paper are summarized as follows:

- To the best of our knowledge, the scenario, where the DF relay using SWIPT helps the primary source and parasitic AmBC source to forward information for their corresponding destinations, is investigated for the first time.
- We achieve the analytical expressions of outage probabilities and throughput of both primary and parasitic relaying links. Via the derived theoretical results, we also discuss the effects of the system parameters on the whole system performance.
- From simulation results, we can see that the considered relaying system has a far larger sum throughput than the one with the only primary relaying link. Besides, it is found that the parasitic relaying link can attain considerable throughput gains at the expense of negligible performance degradation of the primary relaying link. It means that utilizing the provided theoretical results, one can carefully choose the system parameters to drastically improve the system performance of the advised relaying system.

The rest of this paper is structured as follows. Section 2 introduces the transmission scheme, system models and assumptions. In Section 3, the outage probabilities and throughput are derived. Besides, the effects of system parameters are also discussed in Section 3. Numerical and theoretical results are shown in Section 4 to verify our analysis. Finally, we conclude this paper in Section 5.

Notations: P(A) denotes the probability of random event *A*. E{x} is the expectation of random variable *x*. $\mathrm{CN}(\mu ,\sigma^{2})$ stands for the complex Gaussian distribution with mean μ and variance $\sigma^{2}$. Exp(λ) denotes the exponential distribution with mean 1/λ. |x| is the magnitude of complex number *x*.

## 2. System Model

**Figure 1:**
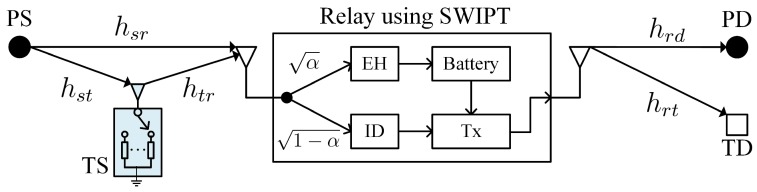
System model.

The considered DF relay network consists of five types of nodes, primary source (PS), tag source (TS), primary destination (PD), tag destination (TD), and relay (R), as illustrated in Figure 1. Due to the possible blockage and severe path loss, PS and TS intend to transmit their information to PD and TD respectively, with the help of DF relay. It is assumed that there is no direct transmission between both source-destination pairs. PS is an active information transmitter while TS, which employs AmBC to convey information, is a passive transmitter. If PS keeps silent, there is no RF signal for TS performing signal reflection. Therefore, the PS-R-PD is the primary relaying link while TS-R-TD is the parasitic relaying link. In practice, PS with stable power supply is usually designed to transmit data with high quality of service (QoS) requirement, e.g., high information rate and reliability. The TS using AmBC always deliveries low rate application, such as measurement data, environment state information or indicator signal [1,2,3,4].

Besides that, the DF relay is not linked to the stable power supply, so that it needs to harvest energy from ambient RF signals. For the PS-R-PD link, $h_{sr}$ and $h_{rd}$ denote the channel coefficients from PT to R and from R to PD, respectively. While, for the parasitic relaying link, $h_{st}$, $h_{tr}$ and $h_{rt}$ stand for the channel coefficients of PS-PT, PT-R, and R-TD, respectively. For clarity, we use $d_{ab}>1$ to denote the transmission distance which corresponds to channel $h_{ab}$; e.g., $d_{sr}$ is the distance from PS to R. The path loss exponent is θ. For convenience, we assume that all channel coefficients follow the independently and identically distributed (i.i.d) complex Gaussian distribution with zero mean and unit variance, e.g., $h_{sr}∼\mathrm{CN}\left(0,1\right)$. That is to say, all channel power gains are i.i.d. exponential distribution with unit mean, e.g., $|h_{sr}{|}^{2}∼\mathrm{Exp}\left(1\right)$. Additionally, all channels experience quasi-static fading and change from block to block.

**Figure 2:**
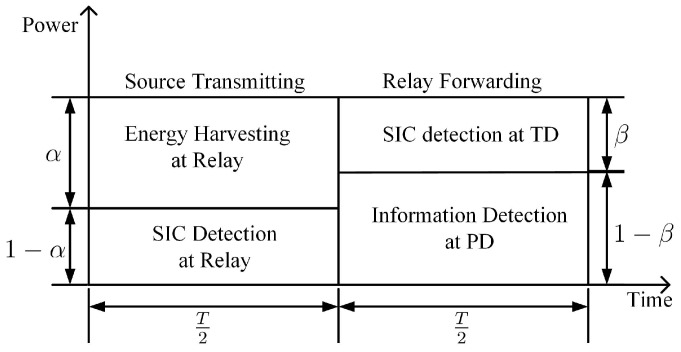
Illustration of transmission time-block.

Since the relay-assisted AmBC is parasitic on the traditional DF relaying transmission, the whole transmission block is still divided into two phases, source transmitting and relay forwarding phases. The relaying block is shown in Figure 2. In order to let the time-block structure compatible with the traditional DF relaying scheme, we herein adopt the power splitting scheme in the DF relaying network. Suppose the whole transmission duration is *T*, we allocate T/2 to the source transmitting phase and the left duration belongs to the relay forwarding phase.

### 2.1. Models in Source Transmitting Phase

In the source transmitting phase, both PS and TS transmit information to the DF relay using SWIPT. In the considered scenario, PS has $L_{s}$ bits to be delivered to PD while TS tries to convey $L_{c}$ bits to TD. In other words, the target information rates of PS and TS are $R_{s}=2L_{s}/T$ and $R_{c}=2L_{c}/T$. Let *s* and *c* be the transmitted baseband symbol from PS and TS respectively. In this paper, both *s* and *c* are generated from Gaussian codebook and ${E\{|s|}^{2}{\}=E\{|c|}^{2}\}=1$. Considering the practical applications, the primary relaying link usually has larger information rate than the parasitic relaying link, i.e., $R_{s}\geq R_{c}$. The received signal at the relay can be expressed as

(1) $$\begin{array}{cc} y_{r} & =\sqrt{P_{s}d_{sr}^{-\theta }}h_{sr}s+\sqrt{P_{s}\eta d_{st}^{-\theta }d_{tr}^{-\theta }}h_{st}h_{tr}sc+n_{r}\\ & =\sqrt{P_{s}d_{sr}^{-\theta }}h_{sr}s+\sqrt{P_{s}\eta d_{t}^{-\theta }}h_{t}sc+n_{r}, \end{array}$$

where $P_{s}$ is the transmit power of PS and 0≤η≤1 is the power reflection coefficient of AmBC. We define $h_{t}=h_{st}h_{tr}$ and $d_{t}^{-\theta }=d_{st}^{-\theta }d_{tr}^{-\theta }$. As $h_{st}∼\mathrm{CN}\left(0,1\right)$ and $h_{tr}∼\mathrm{CN}\left(0,1\right)$, the probability density function (PDF) of $|h_{t}{|}^{2}$ is

(2) $$f_{|h_{t}{|}^{2}}\left(x\right)=2K_{0}\left(\sqrt{y}\right),y\geq 0,$$

where $K_{0}\left(\cdot \right)$ is the zero-order modified Bessel function of the second kind. Note that we omit the reflected antenna noise from the TS for the reason that the reflected noise is degraded by the path loss and becomes negligible [34,45]. $n_{r}$ is the received noise which follows complex Gaussian distribution with zero mean and variance $N_{0}$. The received signal $y_{r}$ is fed into a power splitter with a splitting coefficient α. As Figure 2 $\sqrt{\alpha }y_{r}$ is used to harvest energy (EH) and $\sqrt{\left(1-\alpha \right)}y_{r}$ is employed to perform information detection (ID).

At the energy harvester, the harvested direct current (DC) power is

(3) $$P_{e}=\alpha \rho P_{s}\left(d_{sr}^{-\theta }|h_{sr}{|}^{2}+\eta d_{t}^{-\theta }{\left|h_{t}\right|}^{2}\right),$$

in which 0≤ρ≤1 is the RF-DC power conversion efficiency. Although we can deduce the PDF of $P_{e}$ with the PDFs of $|h_{t}{|}^{2}$ and $|h_{sr}{|}^{2}$, the exact expression of PDF of $P_{e}$ makes performance analyzing intractable. Hence, in this paper, considering the severe path loss and power reflection loss, we alternatively evaluate the performance lower-bound with approximation $P_{e}\approx \alpha \rho P_{s}d_{sr}^{-\theta }{\left|h_{sr}\right|}^{2}$. In practice, ρ depends on the input RF power and appears non-linear behavior with respect to input RF power. While the non-linear power conversion model introduces a considerable complexity when we analyze the statistical performance of wireless-powered communications. As stated in [46], the distribution function of the non-linear energy harvesting (EH) model can be approximately represented by the piecewise linear EH models. Therefore, many works adopt the linear EH model for tractability [44,47,48,49,50]. The DF relay uses all harvested energy to forward its decoded information to PD and TD. Denote the transmit circuit dissipation power with $P_{c}$, then the total energy consumption is $E_{c}=P_{c}T/2$. The available transmit power of the DF relay is given as

(4) $$P_{r}=\max\left\{P_{e}-P_{c},0\right\}.$$

Obviously, $P_{r}=0$ means the relay has no enough harvested energy to forward information.

For information transmission, the first step in the DF relay is to extract information from $\sqrt{\left(1-\alpha \right)}y_{r}$. As the strength of backscattered signal from TS to R is always far less than the primary signal from PS to R due to more severe path loss and signal reflection loss, successive interference cancellation (SIC) is applied to detecting *s* and *c* in sequence. It means that *s* is firstly detected and subtracted from $\sqrt{\left(1-\alpha \right)}y_{r}$. Then *c* is detected from the left information signal. Thus, the received signal-to-interference-plus-noise ratio (SINR) with respect to *s* at relay can be written as

(5) $$\gamma_{r}^{s}=\frac{\left(1-\alpha \right)P_{s}d_{sr}^{-\theta }{\left|h_{sr}\right|}^{2}}{\left(1-\alpha \right)P_{s}\eta d_{t}^{-\theta }{\left|h_{t}\right|}^{2}+N_{0}}.$$

We assume that the ideal channel coding is utilized to protect the information bits. That is to say if $\gamma_{r}^{s}\geq \gamma_{\mathrm{th}}^{s}$ where $\gamma_{\mathrm{th}}^{s}=2^{R_{s}}-1$, the relay can recover *s* successfully. Otherwise, relay cannot obtain *s* without error. Note that if the detected *s* is in error, we omit the probability of detecting *c* successfully in this case, because the detection error of *s* is propagated to the detector of *c* and the success probability of detecting *c* becomes trivial [8]. Consequently, if $\gamma_{r}^{s}<\gamma_{\mathrm{th}}^{s}$, the relay does not forward information and the transmission block ends. When *s* is detected successfully, the received signal-to-plus-noise ratio (SNR) of *c* at relay is

(6) $$\gamma_{r}^{c}=\frac{\left(1-\alpha \right)P_{s}\eta d_{t}^{-\theta }{\left|h_{t}\right|}^{2}}{N_{0}}.$$

Similarly, the detection SNR threshold of *c* is $\gamma_{\mathrm{th}}^{c}=2^{R_{c}}-1$. If $\gamma_{r}^{c}\geq \gamma_{\mathrm{th}}^{c}$, we can recover *c* with no error.

### 2.2. Models in Relay Forwarding Phase

By the DF relaying scheme, the relay discards the received message with error and only forwards the information decoded successfully. Therefore, the DF relay acts in one of two cases, only forwarding *s* and forwarding *s* and *c* simultaneously. If the relay does not forward information, the harvested energy is stored in the battery to maintain the system operation and routine. Next, we describe both relaying cases separately.

In the case of only forwarding *s*, case I, the relay uses $P_{r}$ to transmit *s* to PD. Then, the received signal at PD is

(7) $$y_{d}=\sqrt{P_{r}d_{rd}^{-\theta }}h_{rd}s+n_{d},$$

in which $n_{d}$ is the receiver noise following complex Gaussian distribution with zero mean and variance $N_{0}$. In this case, only PD needs to detect *s* from $y_{d}$. The SNR on *s* at PD is

(8) $$\gamma_{d}^{s}=\frac{P_{r}d_{rd}^{-\theta }{\left|h_{rd}\right|}^{2}}{N_{0}}.$$

While if both *s* and *c* are detected correctly, case II, the relay has the ability of forwarding *s* and *c* to PD and TD. In order to be compatible with the traditional DF relaying scheme, the relay simultaneously transmits *s* and *c* with transmit power $P_{r}$ in the second phase. Let 0≤β≤1, then $\sqrt{1-\beta }s+\sqrt{\beta }c$ is forwarded by the relay. Hence the received signal at PD is

(9) $$y_{d}=\sqrt{\left(1-\beta \right)P_{r}d_{rd}^{-\theta }}h_{rd}s+\sqrt{\beta P_{r}d_{rd}^{-\theta }}h_{rd}c+n_{d}.$$

Similarly, the received signal at TD is

(10) $$y_{t}=\sqrt{\left(1-\beta \right)P_{r}d_{rt}^{-\theta }}h_{rt}s+\sqrt{\beta P_{r}d_{rt}^{-\theta }}h_{rt}c+n_{t},$$

where $n_{t}$ is the receiver noise and $n_{t}∼\mathrm{CN}\left(0,N_{0}\right)$. Considering $L_{x}\geq L_{c}$, we set (1−β)≥β to guarantee the QoS priority of the primary relaying link. As a result, PD just employs traditional detector to extract *s* from $y_{d}$. So, the SINR at PD is

(11) $$\gamma_{d}^{s}=\frac{\left(1-\beta \right)P_{r}d_{rd}^{-\theta }{\left|h_{rd}\right|}^{2}}{\beta P_{r}d_{rd}^{-\theta }{\left|h_{rd}\right|}^{2}+N_{0}}.$$

Differently, the TD has to adopt SIC detection to decode *c* from $y_{t}$. The SINR of *s* at TD is

(12) $$\gamma_{t}^{s}=\frac{\left(1-\beta \right)P_{r}d_{rt}^{-\theta }{\left|h_{rt}\right|}^{2}}{\beta P_{r}d_{rt}^{-\theta }{\left|h_{rt}\right|}^{2}+N_{0}}.$$

If $\gamma_{t}^{s}\geq \gamma_{\mathrm{th}}^{s}$, then *s* can be successfully decoded by TD and subtracted from $y_{t}$. Consequently, the SNR of *c* at TD is expressed as

(13) $$\gamma_{t}^{c}=\frac{\beta P_{r}d_{rt}^{-\theta }{\left|h_{rt}\right|}^{2}}{N_{0}}.$$

While if $\gamma_{t}^{s}<\gamma_{\mathrm{th}}^{s}$, we also deem that both *s* and *c* cannot be decoded without error.

## 3. Outage Performance Analysis

In this section, we investigate the outage performance of the primary PS-R-PD and parasitic TS-R-TD links. As the harvested energy in the source transmitting phase is used to transmit information in the relay forwarding phase, the two hops in DF relaying scheme are not independent any more. Besides, the parasitic AmBC link affects the primary link. Therefore, we take two hops as a whole to analyze the outage probabilities.

### 3.1. Outage Probability of Primary Relay Transmission

For the primary relay transmission, successfully transmitting *s* via PS-R-PD link needs to simultaneously meet three conditions: (1) Relay decodes *s* without error; (2) Relay harvests enough energy, i.e., $P_{r}>0$; (3) PD decodes *s* correctly. Furthermore, as stated in the system model section there are two cases in the relay forwarding phase, case I (only forwarding *s*) and case II (forwarding *s* and *c* simultaneously), according to the results of SIC detection at the DF relay. Let $P_{\mathrm{suc},I}^{s}$ and $P_{\mathrm{suc},\mathrm{II}}^{s}$ denote the successful transmission probabilities in cases I and II, respectively. The overall outage probability with respect to *s* can be expressed as

(14) $$P_{\mathrm{out}}^{s}=1-P_{\mathrm{suc},I}^{s}-P_{\mathrm{suc},\mathrm{II}}^{s}.$$

Next, we derive the expressions of $P_{\mathrm{suc},I}^{s}$ and $P_{\mathrm{suc},\mathrm{II}}^{s}$ separately.

**Theorem** **1.** *The probability of successful transmission of s via PS-R-PD in case I, where the relay only forwards s to PD, can be expressed as*

(15) $$P_{suc,I}^{s}=\left\{\begin{array}{cc} I_{1}+I_{2}, & \mathrm{if}\;\phi <a_{0}a_{2}+a_{1}\\ I_{2}, & \mathrm{otherwise} \end{array}\right.,$$

*where*

$$\begin{array}{cc} I_{1}= & \frac{a_{0}a_{2}+a_{1}-\phi }{2}\sum_{i=1}^{n}\omega_{i}W\left(\frac{a_{0}a_{2}+a_{1}-\phi }{2}x_{i}+\frac{a_{0}a_{2}+a_{1}+\phi }{2}\right)\sqrt{1-x_{i}},\\ I_{2}= & \left[1-2\sqrt{a_{2}}K_{1}\left(2\sqrt{a_{2}}\right)\right]\frac{e^{-\frac{E_{c}}{b_{0}}}}{b_{0}}\left[2\sqrt{b_{1}b_{0}}K_{1}\left(2\sqrt{\frac{b_{1}}{b_{0}}}\right)-\right.\\ & \left.\frac{b_{0}\xi -E_{c}}{2}\sum_{i=1}^{n}\omega_{i}V\left(\frac{b_{0}\xi -E_{c}}{2}x_{i}+\frac{b_{0}\xi -E_{c}}{2}\right)\sqrt{1-x_{i}}\right], \end{array}$$

*where $x_{i}=\cos\left(\frac{2i-1}{2n}\pi \right)$, and $\omega_{i}=\frac{\pi }{n}$. The definitions of $a_{0}$, $a_{1}$, $a_{2}$, $b_{0}$, $b_{1}$, ϕ, W(·) and V(·) can be found in *(A3)*, *(A4)*, *(A9)*, and *(A13)* in Appendix A.*

**Proof.**  See Appendix A.  □

**Theorem** **2.** *The probability of successful transmission of s via PS-R-PD in case II, where the relay simultaneously forwards s and c to PD and TD, can be expressed as*

(16) $$P_{\mathrm{suc},II}^{s}=\left\{\begin{array}{cc} 2\sqrt{a_{2}}K_{1}\left(2\sqrt{a_{2}}\right)G\left(b_{2}\right)-F\left(b_{2}\right), & \mathrm{if}\;0\leq \beta \leq \frac{1}{2^{R_{s}}},\\ 0, & \mathrm{otherwise}. \end{array}\right.$$

*where G(·) and F(·) are as defined in *(A22)* in Appendix B.*

**Proof.**  See Appendix B.  □

Due to Theorem 1 and 2, the overall outage probability with respect to *s* can be deduced. In addition, the throughput of the primary PS-R-PD link is given by

(17) $$\kappa_{s}=\frac{1}{T}L_{s}\left(1-P_{\mathrm{out}}^{s}\right)=\frac{1}{2}R_{s}\left(1-P_{\mathrm{out}}^{s}\right).$$

### 3.2. Outage Probability of Parasitic AmBC Transmission

In contrast to the primary relay transmission, correctly transmitting *c* through the parasitic AmBC transmission has four conditions: (1) Relay decodes *c* correctly; (2) Relay harvests enough energy, i.e., $P_{r}>0$; (3) TD decodes *s* with no error; (4) TD decodes *s* successfully. Accordingly, the overall outage probability of *c* can be written as

(18) $$P_{\mathrm{out}}^{c}=1-P_{\mathrm{suc}}^{c},$$

where

(19) $$P_{\mathrm{suc}}^{c}=P\left(\gamma_{r}^{s}\geq \gamma_{\mathrm{th}}^{s},\gamma_{r}^{c}\geq \gamma_{\mathrm{th}}^{c},\gamma_{d}^{s}\geq \gamma_{\mathrm{th}}^{s},\gamma_{d}^{c}\geq \gamma_{\mathrm{th}}^{c},P_{r}>0\right).$$

**Theorem** **3.** *The probability of successful transmission of c via TS-R-TD can be expressed as*

(20) $$P_{\mathrm{suc}}^{c}=2\sqrt{a_{2}}K_{1}\left(2\sqrt{a_{2}}\right)G\left(\varphi \right)-F\left(\varphi \right),$$

*where the definition of φ can be found in *(A27)* in Appendix C.*

**Proof.**  See Appendix C.  □

Therefore, the throughput of the parasitic TS-R-TD link can be presented as

(21) $$\kappa_{c}=\frac{1}{T}L_{c}\left(1-P_{\mathrm{out}}^{c}\right)=\frac{1}{2}R_{c}\left(1-P_{\mathrm{out}}^{c}\right).$$

### 3.3. Effects of Parameters

After deriving the outage probability and throughput of two relaying links, we can now investigate the effects of the system parameters on transmission performance. By the derived outage probabilities, all parameters affect the performance of $P_{\mathrm{out}}^{s}$ and $P_{\mathrm{out}}^{c}$ greatly. Note that ρ stands for the efficiency of RF-DC power converting. Therefore, it is usually preferable to let ρ approach 1 as closely as possible. In practical scenarios, ρ is determined by the rectifier. Next, therefore, we discuss the effects of these three parameters (α, β, η) on both relaying links.

#### 3.3.1. Effects of α

As α is involved in $a_{1}$, $a_{2}$ and $b_{0}$, there exists great difficulty in investigating the effects of α in a theoretical way. Unavoidably, we have to apply numerical methods to determine the optimal α in this paper. In spite of this, we can explore the asymptotic performance in the extreme cases of α. If α→0, we have $b_{0}\to 0$ and ϕ→∞. Accordingly, there are $P_{\mathrm{suc},I}^{s}=I_{2}\to 0$ and $P_{\mathrm{suc}}^{c}\to 0$. The reason is that the relay cannot harvest any energy on the condition of α=0. On the contrary, if α→1, both $a_{1}$ and $a_{2}$ converge to infinity. Due to (A2) and (A17), in this case it is easy to know $P_{\mathrm{suc},I}^{s}\to 0$, $P_{\mathrm{suc},\mathrm{II}}^{s}\to 0$, and $P_{\mathrm{suc}}^{c}\to 0$. The phenomenon can be interpreted as the fact that no information is passed to relay in this case. As a result, we need to choose an optimal α to minimize the outage probability via numerical methods.

#### 3.3.2. Effects of β

Obviously, β just affects the system performance during the relay forwarding phase and is only involved in $b_{2}$ [cf. (A3)]. For PD, it is expected that the relay allocates as much power as possible to *s*. So $P_{\mathrm{suc},\mathrm{II}}^{s}$ is a monotonically decreasing function with respect to β and the optimal choice is β=0.

However, TD should detect *s* first and detect *c* after subtracting *s*. Thus, we should balance the power allocated to *s* and *c* in order to maximize $P_{\mathrm{suc}}^{c}$. Based on (A26), we can rewrite $P_{\mathrm{suc}}^{c}$ as

(22) $$P_{\mathrm{suc}}^{c}=\left\{\begin{array}{cc} \int_{\xi }^{\infty }\exp\left(-x-\frac{\gamma_{s}N_{0}T}{2d_{rt}^{-\theta }\left(1-\beta -\beta \gamma_{s}\right)\left(b_{0}x-E_{c}\right)}\right)\Theta \left(x\right)dx, & \mathrm{if}\;\beta \in \left[{\left(\frac{\gamma_{\mathrm{th}}^{s}}{\gamma_{\mathrm{th}}^{c}}+\gamma_{\mathrm{th}}^{s}+1\right)}^{-1},{\left(\gamma_{\mathrm{th}}^{s}+1\right)}^{-1}\right)\\ \int_{\xi }^{\infty }\exp\left(-x-\frac{\gamma_{c}N_{0}T}{2d_{sr}^{-\theta }\beta \left(b_{0}x-E_{c}\right)}\right)\Theta \left(x\right)dx, & \mathrm{if}\;\beta \in \left[0,{\left(\frac{\gamma_{\mathrm{th}}^{s}}{\gamma_{\mathrm{th}}^{c}}+\gamma_{\mathrm{th}}^{s}+1\right)}^{-1}\right) \end{array}\right.$$

where $\Theta \left(x\right)=2\sqrt{a_{2}}K_{1}\left(2\sqrt{a_{2}}\right)-2\sqrt{\frac{x-a_{1}}{a_{0}}}K_{1}\left(2\sqrt{\frac{x-a_{1}}{a_{0}}}\right).$ We can see that if $\beta \in \left[{\left(\frac{\gamma_{\mathrm{th}}^{s}}{\gamma_{\mathrm{th}}^{c}}+\gamma_{\mathrm{th}}^{s}+1\right)}^{-1},{\left(\gamma_{\mathrm{th}}^{s}+1\right)}^{-1}\right)$, $P_{\mathrm{suc}}^{c}$ is a monotonically decreasing function of β. Differently, if $\beta \in \left[0,{\left(\frac{\gamma_{\mathrm{th}}^{s}}{\gamma_{\mathrm{th}}^{c}}+\gamma_{\mathrm{th}}^{s}+1\right)}^{-1}\right)$, $P_{\mathrm{suc}}^{c}$ increases as β increasing. That is to say the optimal β to maximize $P_{\mathrm{suc}}^{c}$, i.e., to minimize $P_{\mathrm{out}}^{c}$, is

(23) $$\beta^{⋆}={\left(\frac{\gamma_{\mathrm{th}}^{s}}{\gamma_{\mathrm{th}}^{c}}+\gamma_{\mathrm{th}}^{s}+1\right)}^{-1}={\left(\frac{2^{R_{s}}-1}{2^{R_{c}}-1}+2^{R_{s}}\right)}^{-1}.$$

Interestingly, we can see that $\beta^{⋆}$ is independent of α. It means even if α affects the performance of $P_{\mathrm{out}}^{c}$, it does not influence $\beta^{⋆}$. Oppositely, it is apparent that α influences not only the source transmitting phase but also the relay forwarding phase. So, seeking the optimal α to minimize $P_{\mathrm{out}}^{c}$, we should concern β as β affects the performance of the relay forwarding phase. While, for $P_{\mathrm{out}}^{s}$, the optimal β equals to zero (note that we let β≠0 in practice). It means the optimal α has no relation to the optimal β to maximize $P_{\mathrm{out}}^{s}$.

#### 3.3.3. Effects of η

Similarly, the expressions of the outage probabilities are so complicated with respect to η, thus we have to apply numerical method to investigate the effects of η on the system performance. There is an evident fact that given all other parameters, η=0 makes $P_{\mathrm{out}}^{s}$ achieve the minimum. This is because all harvested power is used to forward *s* to PD and there is no interference. For *c*, increasing η from zero, the strength of the signal reflected by TS becomes greater and it may improve the probability of decoding *c* without error at the relay. Nonetheless, increasing η also causes a degradation of the probability of decoding *s* successfully at the relay. As a result, enlarging η is not always the best choice to minimize $P_{\mathrm{out}}^{c}$. We need to find the optimal η via numerical solutions.

## 4. Simulation

In this section, we show the simulation results to verify our theoretical analysis. The system parameters in simulations follow the default values listed in Table 1, unless otherwise specified. The simulation results are generated by Monte Carlo experiments over $10^{6}$ channel realizations. The theoretical results are obtained by the expressions of the derived outage probabilities. In the figure legends, ‘Sim.’ and ‘The.’ are the abbreviations of simulational and theoretical, respectively.

**Figure 3:**
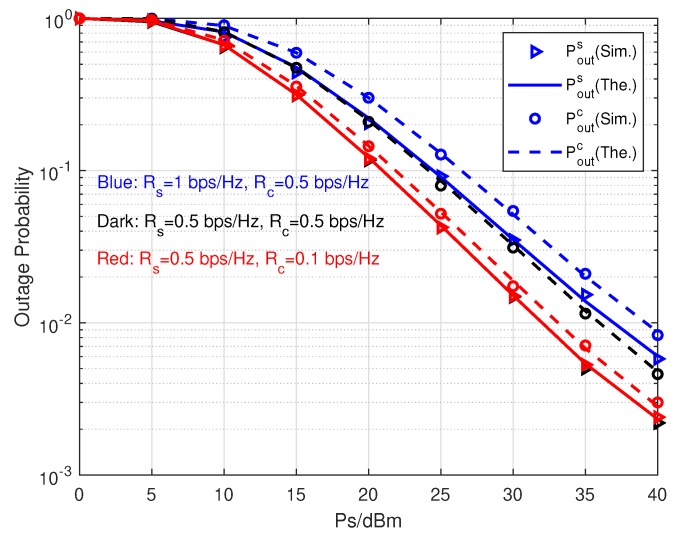
Outage probabilities versus $P_{s}$ with different information rates.

In Figure 3, the outage probabilities are demonstrated with various information rate configurations. First of all, we can see that all theoretical results fit the simulational results closely. It means our derived expressions of outage probabilities can represent the performance of both primary and parasitic relaying links. As $P_{s}$ raises up, both $P_{\mathrm{out}}^{s}$ and $P_{\mathrm{out}}^{c}$ constantly decrease. Moreover, under the same rate configuration $P_{\mathrm{out}}^{s}$ is always less than $P_{\mathrm{out}}^{c}$ given a $P_{s}$. We consider three information rate configurations in this simulation. For $R_{c}=0.5$ bps/Hz, decreasing $R_{s}$ from 1 bps/Hz to 0.5 bps/Hz diminishes not only $P_{\mathrm{out}}^{s}$ but also $P_{\mathrm{out}}^{s}$. Nevertheless, for $R_{s}=0.5$ bps/Hz, decreasing $R_{c}$ from 0.5 bps/Hz to 0.1 bps/Hz just reduces $P_{\mathrm{out}}^{c}$. This phenomenon shows $R_{s}$ plays a more significant role in transmission performance than $R_{c}$. The reason is that the precondition of decoding *c* successfully is that *s* has been decoded without error. Furthermore, the scenario $R_{s}=R_{c}=0.5$ bps/Hz has the maximum performance gap between $P_{\mathrm{out}}^{s}$ and $P_{\mathrm{out}}^{c}$ among all three scenarios. While the scenario with $R_{s}=0.5$ bps/Hz and $R_{c}=0.1$ bps/Hz achieves the minimum performance gap. In other words, the larger the rate difference between primary and parasitic relaying links is, the less the outage probability gap is. Therefore, the derived theoretical results provide useful tools for the system designers to determine system parameters according to the required transmission QoS.

**Figure 4:**
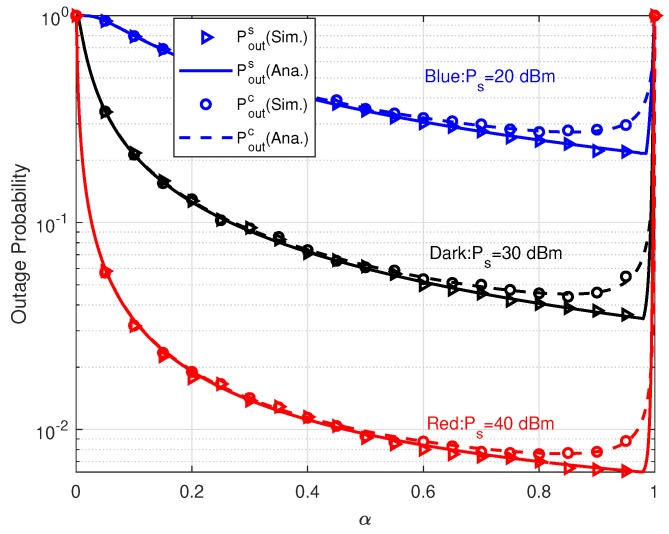
Outage probabilities versus α with different $P_{s}$.

The effects of power splitting coefficient α on outage probabilities are illustrated in Figure 4. Obviously, the simulation results verify our theoretical results well. Given an α, the larger transmit power $P_{s}$ brings in lower outage probability. When α=0, the relay can not harvest any RF energy and has to stop forwarding information. So the outage probabilities equal to 1. As α increasing, the relay harvests more and more energy to forward information during the relay forwarding phase. Thus, $P_{\mathrm{out}}^{s}$ and $P_{\mathrm{out}}^{c}$ start to decrease. When α≤0.6, $P_{\mathrm{out}}^{s}$ and $P_{\mathrm{out}}^{c}$ achieve almost the same performance. While, when α>0.6, the performance gap between $P_{\mathrm{out}}^{s}$ and $P_{\mathrm{out}}^{c}$ gradually appears. The reason is R can simultaneously successfully decode *s* and *c* in most cases when α<0.6. So, $P_{\mathrm{out}}^{s}$ and $P_{\mathrm{out}}^{c}$ have nearly the same values. However, when α>0.6, increasing α lowers the growth of probability of decoding *s* successfully at R and produces more harm to decoding *c* successfully due to the SIC detector. As s result, the outage gap appears. We also can see that given $P_{s}$, $P_{\mathrm{out}}^{c}$ apparently has the minimum value over α∈[0,1]. As for $P_{\mathrm{out}}^{s}$, the optimal α generating minimum $P_{\mathrm{out}}$ approaches 1 extremely closely, e.g., the optimal α is about 0.98 when $P_{s}=40$ dBm. This is because the extremely high α produces large harvested energy and nearly no *c* can be received successfully at R. In this case, although the probability of decoding *s* correctly becomes lower, R could own large transmit power to forward *s* to PD with almost no interference. Given that in the source transmitting phase *s* and *c* always interference each other, it is obviously to deduce that the optimal α tends to allocate more energy to the R-PD transmission without interference.

That is to say that $P_{\mathrm{out}}^{s}$ and $P_{\mathrm{out}}^{c}$ own different optimal power splitting coefficients. When α=1, both $P_{\mathrm{out}}^{s}$ and $P_{\mathrm{out}}^{c}$ converge to 1 again because of information link interruption. Then our analysis on the effect of α is also verified. Comparing these three scenarios with different $P_{s}$, it is observed that the optimal α occurs at the same $P_{s}$ and it is independent on $P_{s}$. From Figure 4, it is concluded that we have to carefully choose α to balance the performance of primary and parasitic relaying links. In Table 1, we let α=0.9 to make both $P_{\mathrm{out}}^{s}$ and $P_{\mathrm{out}}^{c}$ achieve favorable performance.

**Figure 5:**
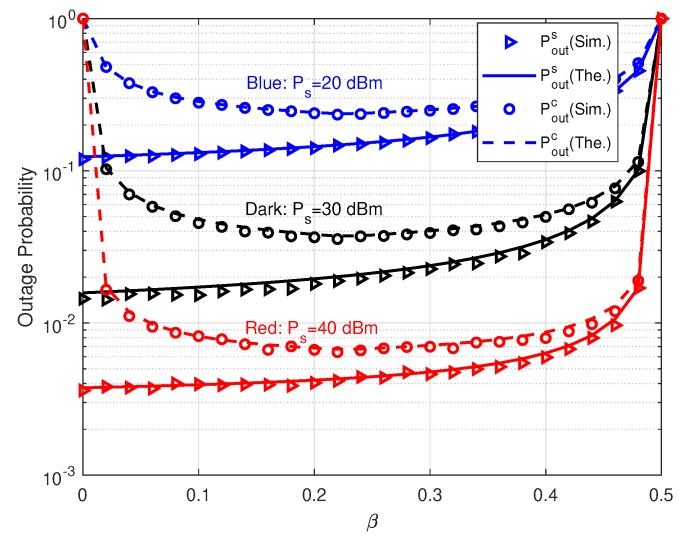
Outage probabilities versus β with different $P_{s}$.

In Figure 5, we show the performance of $P_{\mathrm{out}}^{s}$ and $P_{\mathrm{out}}^{c}$ versus power allocation coefficient β. In this simulation, we set $R_{s}=1$ bps/Hz and $R_{c}=0.5$ bps/Hz. Therefore, by (23) the optimal power allocation coefficient is $\beta^{⋆}\approx 0.22$ for $P_{\mathrm{out}}^{c}.$ Then, the feasible region of β is [0,0.5). The curves of $P_{\mathrm{out}}^{c}$ in Figure 5 also show that β=0.22 makes minimum $P_{\mathrm{out}}^{c}$. On the contrary, $P_{\mathrm{out}}^{s}$ increases as β rises. These results verify our analysis on the effects of β. When β=0, $P_{\mathrm{out}}^{s}$ reaches the minimum value because all relay transmit power $P_{r}$ is allocated to *s*. Differently, if β=1, although total $P_{r}$ is used to forward *c*, $P_{\mathrm{out}}^{c}$ still converges to 1. The reason is that SIC detector at TD cannot decode *c* successfully under the condition of $P_{\mathrm{out}}^{s}=1$. By this figure, it is shown that β also affects $P_{\mathrm{out}}^{s}$ and $P_{\mathrm{out}}^{c}$ greatly and we need to adjust β to coordinate the performance of both relaying links. In practice, the QoS requirement of primary relaying link should be satisfied as far as possible. With the primary QoS constraint, the outage probability of parasitic relaying link could be minimized through optimizing β. For example, in the scenario $P_{s}=30$ dBm, if the constraint is $P_{\mathrm{out}}^{s}\leq 0.02$, the optimal β is 0.22 to minimize $P_{\mathrm{out}}^{c}$. While, if we let $P_{\mathrm{out}}^{s}\leq 0.03$, β should be larger than 0.36. Therefore, the optimal β for $P_{\mathrm{out}}^{c}$ is 0.36, since $\beta^{⋆}$ is out of the feasible range.

**Figure 6:**
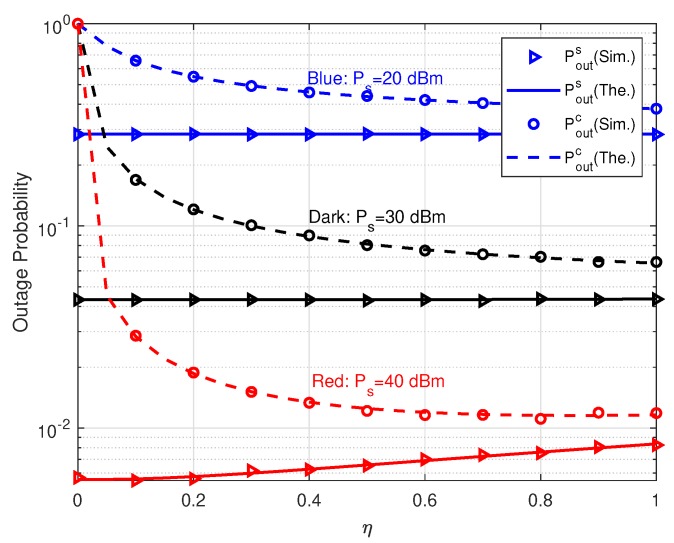
Outage probabilities versus η with different $P_{s}$.

The effects of reflection coefficient η on outage probabilities are investigated in Figure 6. It is notable that $P_{\mathrm{out}}^{c}$ decreases and $P_{\mathrm{out}}^{s}$ nearly keep constant as η increases in the cases of $P_{s}=20$ dBm and $P_{s}=30$ dBm. This is because the reflected signal by TS interferes with the primary link slightly. Therefore, in both cases, we can reduce $P_{\mathrm{out}}^{c}$ via enhancing η while bringing in trivial impact on $P_{\mathrm{out}}^{s}$. In other words, the performance of parasitic relaying link can be improved at the expense of negligible performance degradation of primary relaying link. However, the aforementioned conclusion does not hold if $P_{s}=40$ dBm. We can see that as η increases $P_{\mathrm{out}}^{s}$ becomes larger and larger. The reason is the power of reflection signal conveying *c* is proportional to $P_{s}\eta$. When $P_{s}$ and η are large enough, e.g., $P_{s}=40$ dB and η>0.1, the signal reflected by TS incurs considerable interference to the primary link. Therefore, increasing η increases $P_{\mathrm{out}}^{s}$ in the case of $P_{s}=$40 dB. Furthermore, the increasing $P_{\mathrm{out}}^{s}$ finally enlarges $P_{\mathrm{out}}^{c}$ a little. It is also shown we can choose a suitable η to meet the QoS requirements of primary and parasitic relaying links.

**Figure 7:**
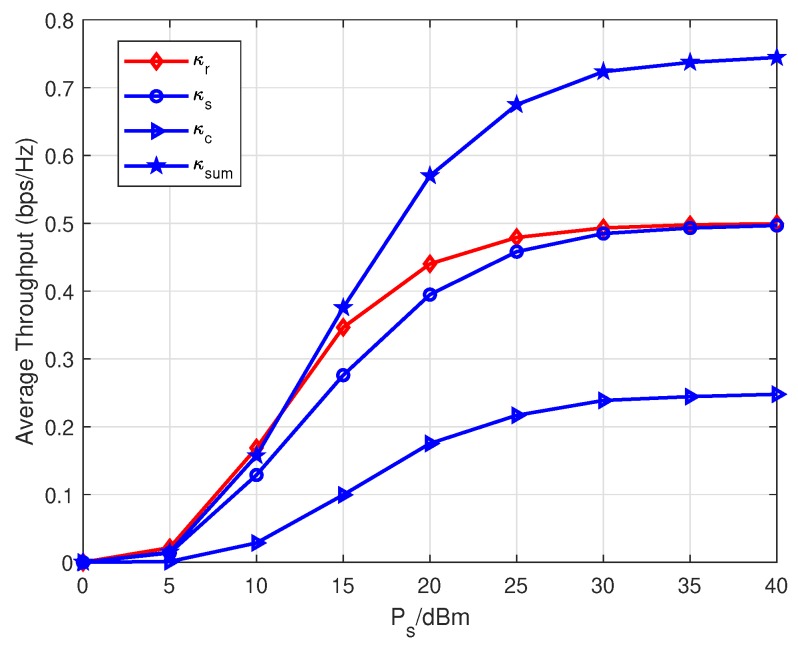
Average throughput versus $P_{s}$.

In Figure 7, average throughput versus $P_{s}$ are depicted. Note that $\kappa_{r}$ stands for the average throughput of the system with only a primary relaying link, i.e., η=0 and β=0. Here, $\kappa_{\mathrm{sum}}$ is the sum throughput, i.e., $\kappa_{\mathrm{sum}}=\kappa_{s}+\kappa_{c}$. Herein we draw $\kappa_{r}$ and $\kappa_{\mathrm{sum}}$ to demonstrate the throughput gain and advantage of the parasitic relaying link. As $P_{s}$ increases, all four types of throughput increase and approach corresponding performance limits. Observing the throughput performance over $P_{s}\in \left[5\mathrm{dBm},35\mathrm{dBm}\right]$, the considered parasitic relaying link can gain certain throughput at the expense of sacrificing a little throughput of the primary relaying link. It is worth pointing out that the sum throughput $\kappa_{\mathrm{sum}}$ outperforms $\kappa_{r}$ when $P_{s}>10$ dBm. That is to say, the parasitic relaying link can attain throughput gains than the traditional SWIPT relaying system which just forwards *s*. Not only that, when $P_{s}>35$ dBm, $\kappa_{s}$ approaches $\kappa_{r}$ closely. It means the throughput degradation caused by parasitic relaying link can be eliminated in the high transmit power scenario and the parasitic relaying link can gain considerable throughput at no additional expense.

## 5. Conclusions

In conclusion, we have studied AmBC, which is parasitic on a relay system that employs simultaneous wireless information and power transfer and decode-and-forward. The relay is responsible for forwarding information both for the primary source and for the AmBC tag source. Both the relay and the tag destination perform successive interference cancellation to eliminate the interference from the primary source and detect the message from tag source. We presented a careful analysis of the outage probabilities of both the primary and parasitic relaying transmissions. The analytical expressions obtained to enable a system designer to perform optimization on the system parameters. Specifically, we have studied the effects of three system parameters, namely power splitting coefficient, forwarding power allocation coefficient and the AmBC reflection coefficient, on the system performance. Simulation results show that the considered relay system achieves more sum throughput than the original relay system. It is also found that the parasitic relaying link is able to achieve sizable throughput at the cost of negligible degradation of the relay system without AmBC tag.

## Figures and Tables

**Table 1:** Parameters in simulations.

Symbol	Definition	Default Value
$P_{s}$	PS transmit power	30 dBm
$d_{sr}$	Distance from PS to R	10 m
$d_{st}$	Distance from PS to TS	12 m
$d_{tr}$	Distance from TS to R	5 m
$d_{rd}$	Distance from R to PD	10 m
$d_{rt}$	Distance from R to TD	10 m
ρ	RF-DC power conversion efficiency	1
α	Power splitting coefficient	0.9
β	Power allocation coefficient	0.4
η	Reflection coefficient	0.6
$P_{c}$	Circuit dissipation power	15.8 μW [51]
θ	Path-loss exponential	3
$R_{s}$	Information rate of PS	1 bit/s/Hz
$R_{c}$	Information rate of TS	0.5 bit/s/Hz
*T*	Transmission block length	1 s
$N_{0}$	Receiver noise power	−65 dBm

## Appendix A. Proof of Theorem 1

In the case I, in the light of the system model, we have

(A1) $$P_{\mathrm{suc},I}^{s}=P\left(\gamma_{r}^{s}\geq \gamma_{\mathrm{th}}^{s},\gamma_{r}^{c}<\gamma_{\mathrm{th}}^{c},\gamma_{d}^{s}\geq \gamma_{\mathrm{th}}^{s},P_{r}>0\right).$$

Substituting (5), (6), (8), and (4) into (A1) and after some manipulations, we have

(A2) $$P_{\mathrm{suc},I}^{s}=P\left(|h_{t}{|}^{2}\leq \frac{|h_{sr}{|}^{2}-a_{1}}{a_{0}},|h_{t}{|}^{2}<a_{2},|h_{sr}{|}^{2}\geq \frac{E_{c}}{b_{0}}+\frac{b_{1}}{|h_{rd}{|}^{2}},{\left|h_{sr}\right|}^{2}>\frac{E_{c}}{b_{0}}\right),$$

in which

(A3) $$\begin{array}{cc} a_{0} & =\eta \gamma_{\mathrm{th}}^{s}\frac{d_{t}^{-\theta }}{d_{sr}^{-\theta }}\\ a_{1} & =\frac{\gamma_{\mathrm{th}}^{s}N_{0}}{P_{s}d_{sr}^{-\theta }\left(1-\alpha \right)}\\ a_{2} & =\frac{\gamma_{\mathrm{th}}^{c}N_{0}}{P_{s}\eta d_{t}^{-\theta }\left(1-\alpha \right)}\\ b_{0} & =P_{s}\alpha \rho d_{sr}^{-\theta }\frac{T}{2}\\ b_{1} & =\frac{\gamma_{\mathrm{th}}^{s}N_{0}}{d_{rd}^{-\theta }}\frac{T}{2}. \end{array}$$

Based on (A2), we can see that if $|h_{sr}{|}^{2}\leq \max\left\{\frac{E_{c}}{b_{0}},a_{1}\right\}$, it is obvious that $P_{\mathrm{suc},I}^{s}=0$, which means *s* cannot be transmitted successfully under this condition. Therefore, we just need to investigate the performance under the condition

(A4) $$|h_{sr}{|}^{2}>\phi =:\max\left\{\frac{E_{c}}{b_{0}},a_{1}\right\}.$$

Moreover, comparing $\frac{|h_{sr}{|}^{2}-a_{1}}{a_{0}}$ to $a_{2}$, we can rewrite (A2) as

(A5) $$\begin{array}{cc} P_{\mathrm{suc},I}^{s}= & \underset{I_{1}}{\underset{︸}{P\left(|h_{t}{|}^{2}\leq \frac{|h_{sr}{|}^{2}-a_{1}}{a_{0}},|h_{sr}{|}^{2}>\phi ,|h_{sr}{|}^{2}\leq a_{0}a_{2}+a_{1},{\left|h_{rd}\right|}^{2}\geq \frac{b_{1}}{b_{0}{\left|h_{sr}\right|}^{2}-E_{c}}\right)}}+\\ & \underset{I_{2}}{\underset{︸}{P\left(|h_{t}{|}^{2}<a_{2},|h_{sr}{|}^{2}>\phi ,|h_{sr}{|}^{2}>a_{0}a_{2}+a_{1},{\left|h_{rd}\right|}^{2}>\frac{b_{1}}{p_{0}{\left|h_{sr}\right|}^{2}-E_{c}}\right)}} \end{array}.$$

Nest, we separately give the expressions of $I_{1}$ and $I_{2}$.

For $I_{1}$, if $\phi \geq a_{0}a_{2}+a_{1}$, then $I_{1}=0$. If $\phi <a_{0}a_{2}+a_{1}$, $I_{1}$ can be rewritten by

(A6) $$\begin{array}{cc} I_{1}= & P\left(|h_{t}{|}^{2}\leq \frac{|h_{sr}{|}^{2}-a_{1}}{a_{0}},\phi <|h_{sr}{|}^{2}\leq a_{0}a_{2}+a_{1},{\left|h_{rd}\right|}^{2}\geq \frac{b_{1}}{b_{0}{\left|h_{sr}\right|}^{2}-E_{c}}\right)\\ & =\int_{\phi }^{a_{0}a_{2}+a_{1}}e^{-x}P\left(|h_{t}{|}^{2}\leq \frac{x-a_{1}}{a_{0}}\right)P\left(|h_{rd}{|}^{2}\geq \frac{b_{1}}{b_{0}x-E_{c}}\right)dx \end{array}.$$

Due to $h_{rd}∼\mathrm{CN}\left(0,1\right)$, it is obvious that

(A7) $$P\left(|h_{rd}{|}^{2}\geq \frac{b_{1}}{b_{0}x-E_{c}}\right)=e^{-\frac{b_{1}}{b_{0}x-E_{c}}}$$

Utilizing the PDF of $|h_{t}{|}^{2}$ in (2), we have

(A8) $$P\left(|h_{t}{|}^{2}\leq \frac{x-a_{1}}{a_{0}}\right)=\int_{0}^{\frac{x-a_{1}}{a_{0}}}2K_{0}\left(\sqrt{x}\right)dx=1-2\sqrt{\frac{x-a_{1}}{a_{0}}}K_{1}\left(2\sqrt{\frac{x-a_{1}}{a_{0}}}\right),$$

where $K_{1}\left(\cdot \right)$ the first-order modified Bessel function of the second kind. Substituting (A7) and (A8) into (A6), we can express $I_{1}$ as

(A9) $$\int_{\phi }^{a_{0}a_{2}+a_{1}}\underset{W(x)}{\underset{︸}{e^{-x-\frac{b_{1}}{b_{0}x-E_{c}}}\left(1-2\sqrt{\frac{x-a_{1}}{a_{0}}}K_{1}\left(2\sqrt{\frac{x-a_{1}}{a_{0}}}\right)\right)}}dx.$$

Although (A9) is a definite integral, it is intractable mathematically. Alternatively, we can resort to the *n*-point Chebyshev-Gauss Quadrature [52] to approximately express (A9) as

(A10) $$I_{1}≃\frac{a_{0}a_{2}+a_{1}-\phi }{2}\sum_{i=1}^{n}\omega_{i}W\left(\frac{a_{0}a_{2}+a_{1}-\phi }{2}x_{i}+\frac{a_{0}a_{2}+a_{1}+\phi }{2}\right)\sqrt{1-x_{i}},$$

where $x_{i}=\cos\left(\frac{2i-1}{2n}\pi \right)$, $\omega_{i}=\frac{\pi }{n}$, and *n* controls the exactness of the computed integral.

For $I_{2}$, define $\xi =\max\left\{a_{0}a_{2}+a_{1},\frac{E_{c}}{b_{0}}\right\}$, there is

(A11) $$I_{2}=P\left(|h_{t}{|}^{2}<a_{2}\right)P\left(|h_{sr}{|}^{2}>\xi ,{\left|h_{rd}\right|}^{2}>\frac{b_{1}}{b_{0}{\left|h_{sr}\right|}^{2}-E_{c}}\right).$$

Like (A8), it can be expressed as

(A12) $$P\left(|h_{t}{|}^{2}<a_{2}\right)=1-2\sqrt{a_{2}}K_{1}\left(2\sqrt{a_{2}}\right).$$

Furthermore, we have

(A13) $$\begin{array}{c} P\left(|h_{sr}{|}^{2}>\xi ,{\left|h_{rd}\right|}^{2}>\frac{b_{1}}{b_{0}{\left|h_{sr}\right|}^{2}-E_{c}}\right)\\ \;=\int_{\xi }^{\infty }e^{-x}P\left(|h_{rd}{|}^{2}>\frac{b_{1}}{b_{0}x-E_{c}}\right)dx\\ \;=\int_{\xi }^{\infty }e^{-x-\frac{b_{1}}{b_{0}x-E_{c}}}dx\overset{\left(a\right)}{=}\frac{e^{-\frac{E_{c}}{b_{0}}}}{b_{0}}\int_{b_{0}\xi -E_{c}}^{\infty }e^{-\frac{t}{b_{0}}-\frac{b_{1}}{t}}dt\\ \;\overset{\left(b\right)}{=}\frac{e^{-\frac{E_{c}}{b_{0}}}}{b_{0}}\left[2\sqrt{b_{1}b_{0}}K_{1}\left(2\sqrt{\frac{b_{1}}{b_{0}}}\right)-\int_{0}^{b_{0}\xi -E_{c}}\underset{V(t)}{\underset{︸}{e^{-\frac{t}{b_{0}}-\frac{b_{1}}{t}}}}dt\right]. \end{array}$$

In step (a), we let $b_{0}x-E_{c}=t$ and step (b) refers to ([53], eq. (3.324)). Following also the *n*-point Chebyshev-Gauss Quadrature, we have

(A14) $$\int_{0}^{b_{0}\xi -E_{c}}V(t)dt≃\frac{b_{0}\xi -E_{c}}{2}\sum_{i=1}^{n}\omega_{i}V\left(\frac{b_{0}\xi -E_{c}}{2}x_{i}+\frac{b_{0}\xi -E_{c}}{2}\right)\sqrt{1-x_{i}}.$$

Then, substituting (A12)–(A14) into (A11), we can obtain the expression of $I_{2}$. So there is

(A15) $$P_{\mathrm{suc},I}^{s}=\left\{\begin{array}{cc} I_{1}+I_{2}, & \mathrm{if}\;\phi <a_{0}a_{2}+a_{1},\\ I_{2}, & \mathrm{otherwise}, \end{array}\right.,$$

which is the desired result.

## Appendix B. Proof of Theorem 2

In the case II, like (A1), there is

(A16) $$\begin{array}{cc} P_{\mathrm{suc},\mathrm{II}}^{s}=P & \left(\gamma_{r}^{s}\geq \gamma_{\mathrm{th}}^{s},\gamma_{t}^{c}\geq \gamma_{\mathrm{th}}^{c},\gamma_{d}^{s}\geq \gamma_{\mathrm{th}}^{s},P_{r}>0\right). \end{array}$$

Following the similar derivation in (A2), $P_{\mathrm{suc},\mathrm{II}}^{s}$ can be rewritten as

(A17) $$P_{\mathrm{suc},\mathrm{II}}^{s}=P\left(a_{2}\leq |h_{t}{|}^{2}\leq \frac{|h_{sr}{|}^{2}-a_{1}}{a_{0}},|h_{rd}{|}^{2}\geq \frac{b_{2}}{b_{0}{\left|h_{sr}\right|}^{2}-E_{c}},{\left|h_{sr}\right|}^{2}>\xi \right),$$

where

(A18) $$b_{2}=\frac{\gamma_{\mathrm{th}}^{s}N_{0}}{\left(1-\beta -\beta \gamma_{\mathrm{th}}^{s}\right)d_{rd}^{-\theta }}\frac{T}{2}.$$

Obviously, if $b_{2}<0$, $P_{\mathrm{suc},\mathrm{II}}^{s}=0$. It represents the event that when the relay simultaneously transmits *s* and *c* to PD, *s* cannot be received correctly. This situation leads to energy inefficiency. To avoid such situation, we should choose β to make $b_{2}>0$; i.e., $0\leq \beta \leq \frac{1}{2^{R_{s}}}$. Meanwhile, recall that in the analysis in the case I, there is no extra constraint on β. As the system performance depends on the statistical channel state information (CSI), to make PD receive *s* successfully with a non-vanishing probability in both cases, we force $0\leq \beta \leq \frac{1}{2^{R_{s}}}$ to hold.

Considering $|h_{sr}|∼\mathrm{Exp}\left(1\right)$, $P_{\mathrm{suc},\mathrm{II}}^{s}$ can be further written as

(A19) $$\begin{array}{cc} P_{\mathrm{suc},\mathrm{II}}^{s}=\int_{\xi }^{\infty }e^{-x} & P\left(a_{2}\leq {\left|h_{t}\right|}^{2}\leq \frac{x-a_{1}}{a_{0}}\right)P\left(|h_{rd}{|}^{2}\geq \frac{b_{2}}{b_{0}x-E_{c}}\right)dx. \end{array}$$

Since $|h_{rd}{|}^{2}∼\mathrm{Exp}\left(1\right)$, we can obtain

(A20) $$P\left(|h_{rd}{|}^{2}\geq \frac{b_{2}}{p_{0}x-E_{c}}\right)=e^{-\frac{b_{2}}{p_{0}x-E_{c}}}.$$

By the PDF of $|h_{t}{|}^{2}$, we have

(A21) $$P\left(a_{2}\leq {\left|h_{t}\right|}^{2}\leq \frac{x-a_{1}}{a_{0}}\right)=2\sqrt{a_{2}}K_{1}\left(2\sqrt{a_{2}}\right)-2\sqrt{\frac{x-a_{1}}{a_{0}}}K_{1}\left(2\sqrt{\frac{x-a_{1}}{a_{0}}}\right).$$

Therefore, under the constraint $0\leq \beta \leq \frac{1}{2^{R_{s}}}$, the successful transmission probability of *s* in case II can be expressed as in (A22).

(A22) $$\begin{array}{cc} P_{\mathrm{suc},\mathrm{II}}^{s} & =\int_{\xi }^{\infty }e^{-x-\frac{b_{2}}{b_{0}x-E_{c}}}\left(2\sqrt{a_{2}}K_{1}\left(2\sqrt{a_{2}}\right)-2\sqrt{\frac{x-a_{1}}{a_{0}}}K_{1}\left(2\sqrt{\frac{x-a_{1}}{a_{0}}}\right)\right)dx\\ & =2\sqrt{a_{2}}K_{1}\left(2\sqrt{a_{2}}\right)\underset{G(b_{2})}{\underset{︸}{\int_{\xi }^{\infty }e^{-x-\frac{b_{2}}{b_{0}x-E_{c}}}dx}}-\underset{F(b_{2})}{\underset{︸}{\int_{\xi }^{\infty }e^{-x-\frac{b_{2}}{b_{0}x-E_{c}}}2\sqrt{\frac{x-a_{1}}{a_{0}}}K_{1}\left(2\sqrt{\frac{x-a_{1}}{a_{0}}}\right)dx}} \end{array}$$

Due to the derivation from (A13) to (A14), we can deduce

(A23) $$\begin{array}{cc} G(b_{2})≃ & \frac{e^{-\frac{E_{c}}{b_{0}}}}{b_{0}}\left[2\sqrt{b_{2}b_{0}}K_{1}\left(2\sqrt{\frac{b_{2}}{b_{0}}}\right)-\right.\\ & \left.\frac{b_{0}\xi -E_{c}}{2}\sum_{i=1}^{n}\omega_{i}V\left(\frac{b_{0}\xi -E_{c}}{2}x_{i}+\frac{b_{0}\xi -E_{c}}{2}\right)\sqrt{1-x_{i}}\right]. \end{array}$$

Unfortunately, we cannot find an analytical expression of $F(b_{2})$. We can resort to Gauss–Laguerre Quadrature [52] to evaluate $F(b_{2})$, i.e.,

(A24) $$F\left(b_{2}\right)≃e^{\xi }\sum_{i=1}^{n}\nu_{i}e^{-\frac{b_{2}}{b_{0}z_{i}-\xi -E_{c}}}2\sqrt{\frac{z_{i}-\xi -a_{1}}{a_{0}}}\cdot K_{1}\left(2\sqrt{\frac{z_{i}-\xi -a_{1}}{a_{0}}}\right),$$

where nodes $\nu_{i}$ and weights $z_{i}$ can be found in [52].

In summary, if $0\leq \beta \leq \frac{1}{2^{R_{s}}}$, then

(A25) $$P_{\mathrm{suc},\mathrm{II}}^{s}=2\sqrt{a_{2}}K_{1}\left(2\sqrt{a_{2}}\right)G\left(b_{2}\right)-F\left(b_{2}\right),$$

and if $\beta >\frac{1}{2^{R_{s}}}$, $P_{\mathrm{suc},\mathrm{II}}^{s}=0$. Therefore, Theorem 2 is proven.

## Appendix C. Proof of Theorem 3

Substitute the expressions of $\gamma_{r}^{s}$, $\gamma_{r}^{c}$, $\gamma_{d}^{s}$ and $\gamma_{d}^{c}$ into (19), after some manipulations, we have

(A26) $$\begin{array}{cc} P_{\mathrm{suc}}^{c} & =P\left(a_{2}<|h_{t}{|}^{2}<\frac{|h_{sr}{|}^{2}-a_{1}}{a_{0}},|h_{sr}^{2}>\xi ,{\left|h_{rt}\right|}^{2}>\frac{\varphi }{b_{0}{\left|h_{sr}\right|}^{2}-E_{c}}\right)\\ & =\int_{\xi }^{\infty }e^{-x}P\left(a_{2}<{\left|h_{t}\right|}^{2}<\frac{x-a_{1}}{a_{0}}\right)P\left(|h_{rt}{|}^{2}>\frac{\varphi }{b_{0}x-E_{c}}\right)dx\\ & =\int_{\xi }^{\infty }e^{-x-\frac{\varphi }{b_{0}x-E_{c}}}\left(2\sqrt{a_{2}}K_{1}\left(2\sqrt{a_{2}}\right)-2\sqrt{\frac{x-a_{1}}{a_{0}}}K_{1}\left(2\sqrt{\frac{x-a_{1}}{a_{0}}}\right)\right)dx\\ & =2\sqrt{a_{2}}K_{1}\left(2\sqrt{a_{2}}\right)G\left(\varphi \right)-F\left(\varphi \right) \end{array},$$

where

(A27) $$\varphi =\max\left\{\frac{\gamma_{s}N_{0}T}{2d_{rt}^{-\theta }\left(1-\beta -\beta \gamma_{s}\right)},\frac{\gamma_{c}N_{0}T}{2d_{sr}^{-\theta }\beta }\right\}.$$

Therefore, Theorem 3 is proven.
